# Action of Iodoform

**Published:** 1879-09

**Authors:** 


Action of Iodoform.—Hogyes (*Archiv fur Experiment. Pharmakologie*) endeavors to arrive at a permanent settlement of the discrepancies between the statement made by previous inquirers concerning the toxic and narcotic properties of the compound in question; further, to test the statements recently made by Binz with regard to its mode of operation. The following is a summary of the chief results of his inquiry:

1. Iodoform, in adequate doses, is fatal to dogs, cats, and rabbits. Death caused by a gradual paralysis of the circulation and respiration; it is preceded by wasting of the body, but not by convulsions.

2. After death, we find fatty changes in the liver, kidneys, heart, and voluntary muscles. One or two hæmorrhagic extravasations are almost always present in the lower lobes of the lungs.

3. Large doses cause marked drowsiness in the dog and cat; no such effect is witnessed in the rabbit, even after a lethal dose. During the period of somnolencey reflex irritability does not appear to be much interferred with.

4. What changes does iodoform undergo before its absorption? If it is introduced in an undissolved condition, the first step is its solution in whatever fatty matter may be at hand (in the intestines, the oily ingredients of the chyme; in the subcutaneous tissue and the serous cavities, the oily constituents of the tissue juices and serous liquids). The oily solution of iodoform next gives up its iodine to any albuminous principles that may be present; the iodide of albumen thus produced is speedily taken up into the blood while a few minute coagula and colorless oil-globules are left behind.

5. Precisely the same series of changes occurs when a solution of iodine in oil is injected under the skin or into a serous sac.

6. An iodide of albumen prepared by mixing white of egg with a solution of iodine in sodium iodide produces narcotic effects in the cat and dog, just like iodoform; like this, moreover, it fails to produce them in the rabbit.

7. Whether we administer iodoform, iodine dissolved in oil, or iodide of albumen, the iodine is gradually eliminated from the system in combination with the alkali metals. Broadly, we may regard the action of iodoform, locally applied, as equivalent to the prolonged and gradual influence of iodine. Its action on the system after absorption is likewise, in the main, that of iodine, but with some hitherto unexplained peculiarities.—*Lon. Med. Recard.*

